# What are the risk factors for the comorbidity of posttraumatic stress disorder and depression in a war-affected population? a cross-sectional community study in South Sudan

**DOI:** 10.1186/1471-244X-12-175

**Published:** 2012-10-19

**Authors:** Touraj Ayazi, Lars Lien, Arne H Eide, Majok Malek Ruom, Edvard Hauff

**Affiliations:** 1 Institute of Clinical Medicine, Faculty of Medicine, University of Oslo, P.O box 1171, Blindern, Oslo, 0318, Norway; 2 Division of Mental Health and Addiction, Department of Research and Development, Oslo University Hospital, Ulleval, Kirkeveien 166, Building 20, Oslo, 0407, Norway; 3 SINTEF Pb. 124 Blindern, Oslo, 0314, Norway; 4 Wau Teaching Hospital, Western Bahr el Ghazal State, Wau, Ghazal, South Sudan; 5 Center for dual diagnosis, Hospital Innlandet Trust, Ottestad, 2312, Norway

**Keywords:** PTSD, Depression, Comorbidity, Tauma, Post-conflict, Socioeconomic, South Sudan

## Abstract

**Background:**

Limited data exists on the association of war trauma with comorbid posttraumatic stress disorder (PTSD)-depression in the general population of low-income countries. The present study aimed to evaluate socioeconomic and trauma-related risk factors associated with PTSD, depression, and PTSD-depression comorbidity in the population of Greater Bahr el Ghazal States, South Sudan.

**Methods:**

In this cross-sectional community study (n=1200) we applied the Harvard Trauma Questionnaire (HTQ) and MINI International Neuropsychiatric Interview (MINI) to investigate the prevalence of PTSD, depression, and PTSD-depression comorbidity. Multinomial logistic regression analyses were conducted to examine the association between these disorders, previous trauma exposure, sociodemographic, and socioeconomic factors.

**Results:**

PTSD only was found in 331 (28%) and depression only in 75 (6.4%) of the study population. One hundred and twelve (9.5%) of the participants had PTSD-depression comorbid diagnosis. Exposure to traumatic events and socioeconomic disadvantage were significantly associated with having PTSD or PTSD-depression comorbidity but not with depression. Participants with a comorbid condition were more likely to be socioeconomic disadvantaged, have experienced more traumatic events, and showed higher level of psychological distress than participants with PTSD or depression alone.

**Conclusions:**

In individuals exposed to war trauma, attention should be given to those who may fulfill criteria for a diagnosis of both PTSD and depression.

## Background

Posttraumatic stress disorder (PTSD) is highly comorbid with other mental health disorders [1], most commonly with depression [2-4]. The comorbid condition of PTSD-depression is reportedly more severe than isolated PTSD or depression. Individuals with PTSD-depression comorbidity were more likely to have a greater level of disability and more severe symptoms when compared with PTSD only or depression only [5-7] and were at greater risk of attempting suicide compared to those with a diagnosis of depression only [8]. O’Donnell et al. [9] investigated the association between depression, PTSD, and PTSD-depression comorbid condition and various predictive variables for each of the diagnoses among a group of injured survivors of severe traffic accidents. The findings indicated similar patterns of predictors for PTSD and depressive symptoms in longer term (over 12 months) posttrauma. Another study conducted among Latina immigrants in the US [10] examined the association between traumatic event exposure and immigration-related factors on the one hand and depression and PTSD-depression comorbidity on the other hand. Different sets of variables correlated with each of these two conditions. Participants with a higher number of traumatic events were more likely to receive a PTSD-depression comorbid diagnosis.

There has been increasing focus on posttraumatic conditions in war-affected populations during the last decade [11,12], and a few studies have also included data on PTSD-depression comorbidity. Mollica et al. [6] studied Bosnian refugees in a refugee camp and reported a PTSD-depression comorbidity rate of 21%. In a study of Cambodian refugees performed two decades after resettlement in the US, high levels of comorbidity between the two diagnoses associated with exposure to traumatic events was found [13]. In their review of the literature on refugee studies, Fazel et al. [14] reported high levels of PTSD-depression comorbidity.

Various explanations have been proposed on the effect of trauma exposure on mental health. Miller and Rasmussen (2010) [15] distinguish between two different approaches: the trauma-focused approach which emphasizes on exposure to traumatic events as the key factor influencing mental health and various studies have documented the contribution of traumatic events [16,17]. Another main approach is a psychosocial framework which argues that focusing exclusively on trauma exposure is not adequate. Instead, the influence of aggravated social and material conditions related to armed conflict should be emphasized [15,18]. Studies have shown that lower socioeconomic status is associated with higher rate of PTSD [19,20] and depression [21-23] among trauma-exposed individuals. However, most of these studies have been performed in high-income countries. Some of the few studies from low-income countries also confirm the association of socioeconomic disadvantages with greater emotional distress [24,25] or general physical and mental health [26]. Little is known, however, about the prevalence and severity of PTSD-depression comorbid condition, and its socioeconomic risk factors in low-income countries.

Such data are particularly important in countries constructing or reconstructing their health services after war or other violent conflicts. South Sudan is one of the most impoverished countries in the world and health facilities are extremely scant [27]. In addition to economic hardship, the country has experienced a 21-year period of armed conflict. The signing of the Comprehensive Peace Agreement (PCA) in 2005 ended the extensive war-related violence and large-scale forced displacement and resulted in creation of the new state of South Sudan in 2011. Despite this positive pattern of change, the growing influx of returnees to South Sudan has placed an extraordinary strain on already scant services and resources. Previous studies on South Sudanese refugees have indicated high level of trauma exposure and PTSD symptom among this population [28,29]. To our knowledge, only one study [30] on mental health has been carried out in post-conflict South Sudan at the time of our investigation. The study, which was conducted in the capital Juba, reported a high prevalence of PTSD and depression (36.2% and 49.9%, respectively). However, PTSD-depression comorbidity was not investigated and only symptom rating scales were used (rather than diagnostic interview).

In a community study of the mental health of the population of four states in the Greater Bahr el Ghazal region, South Sudan, we examined the prevalence of PTSD-depression comorbidity, PTSD and depression separately, as well as the association between these disorders, previous exposure to traumatic events, and socioeconomic factors.

Our data, collected from rural and urban settings, provide the opportunity to study risk factors for PTSD, depression, and PTSD-depression comorbidity in a population that is very different than those investigated in previous studies of this comorbid condition, and with exposure to complex traumatic events during a longer period of time. This study aimed to examine patterns of risk factors for the three respective conditions in a war exposed population in a low-income country.

This article thus attempted to answer the following questions:

What are the prevalence of PTSD, depression, and PTSD-depression comorbidity?

What are the socioeconomic and trauma-related risk factors for PTSD-depression comorbidity and PTSD and depression individually?

What are the levels of psychological distress associated with PTSD, depression, and PTSD-depression comorbidity?

## Methods

**Figure 1:**
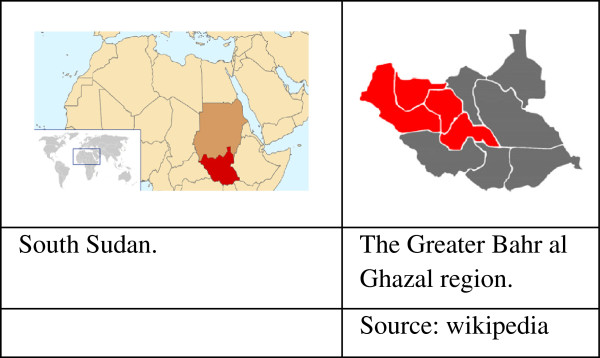
South Sudan and the Greater Bahr al Ghazal states.

A cross-sectional community survey was conducted in the Greater Bahr el Ghazal region of South Sudan in 2010. The Greater Bahr el Ghazal region consists of the following four states: Northern Bahr el Ghazal, Western Bahr el Ghazal, Lakes, and Warrap (Figure 1). It borders the Central African Republic to the west and Sudan to the north and has an estimated population of three million. Major part of the area is covered by swamps and ironstone plateaus. The region is populated by different ethnic groups: Dinka is the major one and other ethnic groups are Blanda, Jur/ Lou, Nuer, Bari and Zande [31]. The population in the region is predominantly rural with some variety within the four states; 92% of the population in Northern Bahr el Ghazal is classified as rural, compared to 57% in Western Bahr el Ghazal. Besides English which is the official language and Arabic which is spoken widely in the region, Dinka, Blanda, Jur/ Lou, Nuer, Bari and Zande are the spoken indigenous languages [31,32].

The sample frame was the general population of the four states in the Greater Bahr el Ghazal region. A multistage random cluster sampling method was used. Nine randomly selected administrative units (‘Boma’) constituted the survey clusters, with a corresponding running cumulative population size for each Boma. The population data were based on the 2008 Sudan census [32]. These data were considered the most accurate population data available. In the next stage, the “spin-the-pen” method from the WHO Expanded Programme on Immunization [33] was used for household selection: the approximate geographic center of the area was identified and one household along an imaginary line connecting the center to the periphery was selected at random. Subsequent households were then selected by visiting every third closest household. Within each selected household, individuals who were 18 years or older and gave informed consent to take part in the study were assigned a number. A card was drawn at random from a deck of cards with corresponding numbers. The randomly selected household member was then interviewed. Individuals who were not able or declined to give informed consent or were visibly intoxicated were excluded from the study.

The participants were interviewed by health personnel (n= 11, five women and six men) from the region who were familiar with the cultural traditions and fluent in relevant local languages. They participated in two rounds of training workshops (9 days) prior to the data collection, during which the interviewers were trained in using the survey instruments. Furthermore, the cultural acceptability of the interview protocol also was discussed. The research instruments were available both in English and Arabic, but the main language used was Arabic which is widely used in the area. In addition, the key terms of the questionnaire were discussed and translated to indigenous languages of the area to ensure that the interviewers could easily explain all the items to the participants. Each household was approached by both a male and a female interviewer to ensure the interviewer’s gender would match that of the participant. In case of identifying any psychopathology with urgent treatment need amongst the participants, the interviewer referred the subject to an associated health provider. A total of 1236 households were contacted from which 1200 participants were recruited. The response rate was 95%. Response rates tend to be particularly high in low-income countries [34-36].

Ethical clearance was obtained from the Research Department in the Ministry of Health of the Government of South Sudan and the Norwegian Regional Committee for Medical Research.

### Instruments

A questionnaire was administered to all participants addressing socio-demographic factors, including sex, age, marital status, level of education, employment situation, monthly household income, and rural–urban setting. Due to the high influx of returnees to the region of study [37], the participants were also asked whether or not he/she was a returnee. A modified version of the United Nations’ definition of returnee [38] was used for the purpose of this study. A returnee was defined as a person who had left his/her place of origin (regardless of the reason), but who has returned to his/her place of origin. The questionnaire also included the Harvard Trauma Questionnaire (HTQ), which is a widely used instrument for assessing history of exposure to traumatic events and PTSD symptom criteria. The HTQ has been adapted for and used in various cultures and languages [39]. The Arabic version of HTQ was employed in this study, after minor adaptations for the specific traumatic events in the South Sudan setting. The HTQ includes 40 questions on exposure to traumatic events with “Yes” or “No” answer choices. In order to facilitate comparison of our findings with those of other relevant studies, 16 questions on exposure to traumatic events from the HTQ were selected. These 16 traumatic events were identical to those applied by Roberts et al.’s [30] in their study of exposure to traumatic events among a population in Juba, Southern Sudan. We also applied the same cut-off points as Roberts et al. for the number of previous and recent traumatic event exposure (cut-off points were eight and four events, respectively) and hence treated exposure to traumatic event as a dichotomized variable. Participants were asked to confirm or disconfirm being exposed to each of these 16 traumatic events: a) during the civil war (from 1983 to 2005) and b) after the Peace Agreement (after 2005). This gave us the opportunity to assess both recent and older traumatic experiences, which may also differ in character.

The HTQ also contains 40 questions aimed at identifying PTSD “caseness”. Participants were asked to report experiencing the symptoms in question on a four-point scale ranging from “not at all”, “a little”, “quite a bit”, to “extremely” within the previous two weeks. The PTSD score was calculated based on the respondents’ sum score on the first 16 items of the HTQ symptoms. A higher score indicates likelihood of suffering from PTSD. A conventional cut-off point of 2.0 was used to identify ‘checklist positive’ for PTSD in accordance with previous studies [30,40-42].

MINI International Neuropsychiatric Interview (MINI) [43] is a structured diagnostic psychiatric interview instrument and has been translated and used in many languages, including Arabic; it has also been applied in various cultures and settings [44]. The Major Depressive Episode section of the MINI (Arabic version) was used to detect major depressive episode (here after referred to as depression).

The General Health Questionnaire (GHQ-28) is a screening instrument widely used to detect psychological distress in community settings and non-psychiatric clinical settings [45]. It has been used in various populations and cultural settings [46] including Sudan [47]. Scores on the GHQ-28 were calculated by summarizing the responses to the 28 questions, applying the Likert scale of scoring (0-1-2-3). Mean scores were calculated; higher score on the GHQ-28 indicates more severe psychological distress (score range, 0–84) [48]. Internal reliability was evaluated using Cronbach-alpha and estimated at 0,93 for MINI (depression), 0.94 for HTQ (PTSD), and 0.94 for GHQ-28 (psychological distress) which all were above the commonly accepted level of 0.70 [49].

### Data analyses

Data analyses were conducted using SPSS (PASW) 18.0. Descriptive statistics were applied to express the characteristics of the sample and the extent of PTSD, depression, and their co-occurrence. The analyses were adjusted for the cluster design.

The dependent variable, diagnostic status, consisted of four diagnostic categories: “depression only” (participant who were classified as having depression but not PTSD), “PTSD only” (participants identified as having PTSD but not depression), “comorbid PTSD-depression” (participants with both depression and PTSD), and “neither PTSD nor depression” (participants who scored negatively on both PTSD and depression criteria). Independent variables included traumatic event exposure (both during the war and after the Peace agreement) and socio-demographic factors (age, sex, marital status, rural–urban setting, income regularity, employment status, education level, household income, and being a returnee). In order to investigate the association between socioeconomic disadvantages and various diagnostic categories, we created a combined variable to represent socioeconomic disadvantages by joining the following five conditions: having no regular income, low monthly income, unemployment, being widowed/separated/living in a polygamous marriage, having no formal education, This combined variable had three levels: “severely disadvantaged” (fulfilling four or five conditions of having no regular income, having low monthly income, being unemployed, being widowed/divorced/polygamous, and having no formal education), “moderate disadvantaged” (two or three of the five conditions are met), and “mildly/not disadvantaged” (one or none of the five conditions were fulfilled).

Bivariate regression analysis was conducted to assess the possible impact of various independent variables, including traumatic event exposure, demographic variables and socioeconomic disadvantage, on the dependent variable (diagnostic status).

Separate multinomial logistic regressions were then applied in order to examine the association of traumatic event exposure, demographic factors, and socioeconomic disadvantage, on the one hand, and the likelihood of belonging to the diagnostic categories of the dependent variable, while controlling for independent variables. These multinomial regressions were used to determine similarities/differences in combination of predictors between 1) having depression only, PTSD only, or comorbid PTSD-depression compared to having “neither PTSD nor depression”; 2) having depression only and PTSD only compared to having a comorbid diagnosis; 3) having depression only compared to having PTSD only.

The multinomial analysis included the combined socioeconomic disadvantage variable as well as age, sex, rural–urban setting, being a returnee, and exposure to traumatic events. These analyses enabled us to examine the influence of the combined variable on having depression only, PTSD only and comorbid condition when all other variables were controlled for.

Severity of psychological distress associated with each of the three diagnostic categories (PTSD only, depression only, and PTSD-depression comorbidity) was measured by one-way ANOVA analysis.

## Results

PTSD diagnosis was found in 448 (37.6%) of participants and 189 (15.9%) fulfilled the diagnostic criteria for major depressive episode. The rate of PTSD only (with absence of a depression diagnosis) was 28.1% (n=331) and the rate of depression only (with absence of a PTSD diagnosis) was 75 (6.4%). Comorbid PTSD-depression was identified in 112 (9.5%) of the total sample (Table 1). Table 1 also shows socio-demographic characteristics of participants across PTSD only, depression only, and PTSD-depression comorbidity groups.

**Table 1:** Socio-demographic characteristics of the study population and differences between various diagnostic categories N (%)

	**Overall N (%)**	**Depression only N (%)bMean 95% CI**	**PTSD only N (%) Mean 95% CI**	**Comorbid N (%) Mean 95% CI**	**Neither PTSD nor depression N (%)**
	1200 (100%)	75 (6.4) 0. (0.05-0.08)	331 (28.1) 0.28 (0.25-0.31)	112 (9.5) 0.09 (0.08-0.11)	662 (56.1)
**Sex**					
Male	645 (56.0)	43 (58.9) 0.07 (0.05-0.09)	168 (52.3) 0.26 (0.22-0.29)	49 (45.0) 0.08 (0.05-0.10)	385 (59.4)
Female	506 (44.0)	30 (41.1) 0.06 (0.04-0.08)	153 (47.7) 0.30 (0.26-0.34)	60 (55.0) 0.11 (0.09-0.14)	263 (40.6)
**Age**					
18-25	308 (25.7)	21 (22.3) 0.06 (0.03-0.8)	51 (22.8) 0.27 (0.22-0.32)	18 (20.2) 0.07 (0.05-0.10)	213 (27.7)
26-35	391 (32.6)	32 (34.0) 0.06 (0.04-0.09)	67 (29.9) 0.30 (0.24-0.33)	29 (32.6) 0.10 (0.07-0.13)	251 (32.6)
36-50	395 (32.9)	31 (33.0) 0.06 (0.04-0.90)	73 (32.6) 0.26 (0.22-0.31)	35 (39.3) 0.11 (0.08-0.14)	249 (32.4)
> 50	89 (7.4)	9 (9.6) 0.10 (0.04-0.17)	22 (9.8) 0.31 (0.22-0.41)	6 (6.7) 0.07 (0.02-0.12)	52 (6.8)
**Marital status**					
Single	320 (26.7)	18 (19.1) 0.05 (0.03-0.08)	49 (21.9) 0.28 (0.23-0.32)	18 (20.2) 0.06 (0.04-0.09)	230 (29.9)
Married (one wife)	559 (46.6)	44 (46.8) 0.63 (0.04-0.9)	111 (49.6) 0.28 (0.25-0.32)	39 (43.8) 0.09 (0.07-0.11)	352 (45.8)
No longer married	81 (6.8)	7 (7.4) 0.05 (0.01-0.11)	19 (8.5) 0.31 (0.22-0.41)	4 (4.5) 0.09 (0.03-0.15)	50 (6.5)
Living in polygamous marriage	215 (17.9)	25 (26.6) 0.09 (0.06-0.13)	38 (17.0) 0.25 (0.20-0.31)	26 (29.2) 0.15 (0.10-0.20)	123 (16.0)
**Education**					
Secondary or higher	387 (32.3)	17 (18.1) 0.04 (0.02-0.06)	57 (25.4) 0.27 (0.22-0.31)	17 (19.1) 0.05 (0.28-0.74)	289 (37.6)
Primary	359 (29.9)	26 (27.7) 0.06 (0.04-0.08)	74 (33.0) 0.30 (0.24-0.33)	25 (28.1) 0.09 (0.06-0.12)	226 (29.4)
Never attended school	434 (36.2)	49 (52.1) 0.09 (0.06-0.11)	90 (40.2) 0.29 (0.25-0.33)	45 (51.6) 0.14 (0.11-0.17)	242 (31.5)
**Employment**					
Paid work	291 (24.3)	18 (19.1) 0.053 (0.03-0.08)	55 (24.6) 0.27 (0.22-0.32)	14 (15.7) 0.06 (0.03-0.08)	196 (25.5)
Self-employment	484 (40.3)	47 (50.0) 0.08 (0.06-0.11)	72 (32.1) 0.24 (0.21-0.29)	33 (37.1) 0.10 (0.07-0.12)	320 (41.6)
Student	144 (12.0)	6 (6.4) 0.04 (0.01-0.10)	24 (10.7) 0.24 (0.14-0.34)	8 (9.0) 0.03 (0.01-0.7)	104 (13.5)
Homemaker	67 (5.6)	4 (4.3) 0.04 (0.01-0.09)	12 (5.5) 0.24 (0.14-0.34)	1 (1.1) 0.03 (0.0-0.07)	50 (6.5)
Retired	23 (1.9)	0 (0.0)-	6 (2.7) 0.24 (0.13-0.35)	2 (2.2) 0.3 (0.0-0.07)	14 (1.8)
Unemployed	111 (9.3)	8 (8.5) 0.54 (0.02-0.10)	40 (17.9) 0.50 (0.40-0.54)	24 (27.0) 0.23 (0.15-0.31)	39 (5.1)
**Household monthly income in US dollars**					
<75	553 (46.1)	40 (42.6) 0.05 (0.03-0.07)	118 (52.7) 0.31 (0.30-0.34)	54 (60.7) 0.12 (0.09-0.15)	330 (42.9)
75-200	209 (17.4)	12 (12.8) 0.50 (0.02-0.08)	35 (15.6) 0.26(0.21-0.32)	11 (12.4) 0.06 (0.03-0.10)	143 (18.6)
200 – 350	85 (7.1)	3 (3.2) 0.35 (0.01-0.08)	18 (8.0) 0.32 (0.23-0.42)	7 (7.9) 0.83 (0.03-0.14)	56 (7.3)
> 350	29 (2.4)	0 (0.0) 0.0	3 (1.3) 0.18 (0.04-0.33)	0 (0.0) 0.0	25 (3.3)
**Socioeconomic Disadvantages**					
Mildly/not disadvantaged	339 (37.3)	13 (3.8) 0.04 (0.02-0.06)	87 (25.7) 0.27 (0.22-0.31)	19 (5.6) 0.06 (0.03-0.80)	220 (64.9)
Moderately disadvantaged	478 (53.3)	35 (7.2) 0.07 (0.05-0.9)	141 (29.0) 0.30 (0.25-0.33)	45 (9.2) 0.09 (0.07-0.12)	266 (54.6)
Severely disadvantaged	87 (9.4)	5 (5.7) 0.05 (0.01-0.09)	28 (32.2) 0.32 (0.22-0.42)	26 (29,9) 0.30 (0.20-0.38)	28 (32.2)
**Returnee**					
No	781 (65.1)	64 (68.1) 0.07 (0.05-0.09)	108 (48.2) 0.23 (0.20-0.26)	45 (50.6) 0.71 (0.05-0.09)	552 (71.8)
Yes	386 (32.2)	26 (27.7) 0.04 (0.02-0.07)	112 (50.0) 0.38 (0.33-0.43)	41 (46.1) 0.14 (0.10-0.17)	200 (26.0)
**Setting**					
Rural	259 (22.2)	36 (38.3)0.14 (0.09-0.18)	43 (19.2) 0.22 (0.17-0.27)	11 (11.8) 0.05 (0.02-0.07)	169 (22.0)
Urban	917 (77.8)	58 (61.7) 0.04 (0.03-0.06)	181 (80.8) 0.30 (0.27-0.33)	78 (88.2) 0.10 (0.09-0.13)	600 (78.0)

Table 2 shows the result of bivariate regression analysis. Several variables were significantly associated with the diagnoses. Socioeconomic disadvantage, rural/urban setting, and having no formal education were the variables which appeared to have an unadjusted impact on all three conditions (depression only, PTSD only, and PTSD-depression comorbidity). Exposure to traumatic events had no unadjusted effect on depression only, but showed significant impact on PTSD only and comorbid condition.

**Table 2:** Unadjusted odds ratio (95% CI): association between various independent variables on depression only, PTSD only, and PTSD-depression comorbidity, compared to having neither PTSD nor depression, unadjusted

	**Unadjusted odds ratio (95% CI)**		
	**Depression only**	**PTSD only**	**Comorbid**
**Sex**			
Male	1	1	1
Female	1.021 (0.624-1.670)	1.333 (1.018-1.745) *	1.793 (1.191-2.697) *
**Age (years)**			
18-25	1	1	1
26-35	1.224 (0.638-2.350)	1.132 (0.798-1.605)	1.537 (0.879-2.689)
36-50	1.180 (0.615-2.263)	1.050 (0.739-1.490)	1.671 (0.966-2.891)
> 50	2.106 (0.882-5.028)	1.358 (0.794-2.324)	1.085 (0.416-2.830)
**Marital status**			
Single	1	1	1
Married	1.281 (0.698-2.350)	1.103 (0.803-1.515)	1.586 (0.917-2.744)
No longer married	1.021 (0.328-3.185)	1.233 (0.710-2.142)	1.519 (0.605-3.816)
Living in polygamous marriage	1.995 (1.000-4.001) *	1.095 (0.725-1.656)	2.856 (1.557-5.239) *
**Education**			
Secondary or higher	1	1	1
Primary	1.732 (0.870-3.449)	1.250 (0.896-1.743)	3.702 (2.140-6.403) *
Never attended school	2.892 (1.544-5.417) *	1.428 (1.035-1.969) *	2.019 (1.107-3.682) *
**Employment**			
Paid work	1 (ref.)	1 (ref.)	1 (ref.)
Self-employment	1.721 (0.906-3.268)	0.981 (0.693-1.387)	1.678 (0.931-3.025)
Student	0.904 (0.335-2.435)	1.293 (0.824-2.029)	1.116 (0.478-2.609)
Homemaker	0.815 (0.225-2.957)	0.812 (0.432-1.524)	0.448 (0.100-2.007)
Retired	0.000	1.000 (0.370-2.702)	2.206 (0.576-8.447)
Unemployed	2.679 (0.950-7.550)	4.250 (2.490-7.253) *	9.559 (4.607-19.835) *
**Regular income**			
Yes	1	1	1
No	1.708 (0.958-3.046)	1.295 (0.963-1.741)	1.573 (0.976-2.534)
**Household monthly income in US dollars**			
<75	1	1	1
75-200	0.772 (0.365-1.634)	0.707 (0.486-1.027)	0.470 (0.239-0.925) *
200 - 350	0.616 (0.180-2.105)	0.957 (0.575-1.595)	0.613 (0.266-1.416)
> 350	0.000	0.362 (0.135-0.971) *	0.000
**Returnee**			
No	1	1	1
Yes	0.905 (0.511-1.603)	2.431 (1.835-3.221) *	2.770 (1.829-4.196) *
**Rural/Urban**			
Rural	1	1	1
Urban	0.349 (0.214-.569)*	1.470 (1.049-2.059) *	2.548 (1.363-4.761)*
Socioeconomic disadvantage**			
Mildly/not disadvantaged	1	1	1
Moderately disadvantaged	2.644 (0.884-7.912)	1.267 (0.923-1.738)	2.016 (1.149-3.535) *
Severely Disadvantaged	2.194 (1.133-4.249) *	2.242 (1.288-3.903) *	9.770 (4.880-13.561) *
**Number of traumatic events during the war**			
<8 events	1	1	1
≥ 8 events	1.232 (0.664-2.283)	3.789 (2.799-5.129) *	4.173 (2.720-6.402)*
**Number of traumatic events after the war**			
<4 events	1	1	1
≥ 4 events	1.004 (0.347-2.908)	4.357 (2.819-6.733) *	1.942 (0.995-3.947)

*p <0.05.

** The combined variable: the combination of having no regular income, having low monthly income, being unemployed, being widowed/divorced/polygamous, and having no formal education.

The result of multinomial analysis indicated that being severely disadvantaged was significantly associated with having a comorbid diagnosis (OR=8.09) or PTSD only (OR=2.34). Socioeconomic disadvantage was not significantly associated with depression only. Similarly, the extent of exposure to traumatic events during the war and after the Peace Agreement were significantly associated with having a PTSD only or comorbid diagnoses when other variables were controlled for, while these showed no significant association with depression only. Participants who had experienced eight or more traumatic events during the war were more likely to be in the PTSD only category or the comorbid category than in either the depression only or the “neither PTSD nor depression” categories. Similarly, participants were more likely to be in the PTSD only category or comorbid category than the depression only or “neither PTSD nor depression” categories if they experienced four or more traumatic evened after the Peace Agreement.

Table 3 also displays the factors distinguishing depression only, PTSD only, or PTSD-depression comorbidity from “neither PTSD nor depression” by comparing the corresponding set of risk factors for each of the diagnostic categories. The impact of the independent variables was similar for PTSD only and comorbid condition. The impact of the independent variables for depression only, however, differed from the other two diagnoses. The only variable that distinguished the depression only group from the “neither PTSD nor depression” group was rural/urban setting: with all other variables controlled for, urban residency seems to have a protective effect on depression only. Having a PTSD only or a comorbid diagnosis was associated with severe socioeconomic disadvantage and exposure to traumatic events during the war and after the Peace Agreement. In addition, being a returnee and residing in urban area were significantly associated with having a PTSD only or a comorbid diagnosis.

**Table 3:** Adjusted odds ratio (95% CI): association between various independent variables on depression only, PTSD only, and PTSD-depression comorbidity, compared with having neither PTSD nor depression, adjusted

	**Adjusted odds ratio (95% CI)**		
	**Depression only**	**PTSD only**	**Comorbid**
**Sex**			
Male	1	1	1
Female	0.652 (0.334-1.274)	1.253(0.878-1.789)	1.479 (0.885-2.470)
**Age**			
18-25	1	1	1
26-35	0.806 (0.361-1.799)	1.150 (0.735-1.799)	1.1573 (0.763-3.242)
36-50	0.685 (0.306-1.536)	.983(0.627-1.540)	1.556 (0.770-3.145)
>50	1.324 (0.428-4.096)	1.693 (0.842-3.407)	1.690 (0.561-5.090)
**Returnee**			
No	1	1	1
Yes	0.916 (0.440-1.909)	1.582 (1.111-2.252) *	1.717 (1.034-2.851) *
**Rural/Urban**			
Rural	1	1	1
Urban	0.284 (0.148-0.543) *	2.894 (1.681-4.984) *	2.910 (1.205-7.026) *
**Socioeconomic Disadvantage**			
Mildly/not disadvantaged	1	1	1
Moderately disadvantaged	1.982 (0.966-4.067)	1.444 (0,923-2.098)	2.117 (0.898-3.929)
Severely Disadvantaged	1.725 (0.441-6.753)	2.341 (1.245-4.402) *	8.096 (3.688-17.774) *
**Number of traumatic events during the war**			
<8 events ≥ 8 events	1	1	1
	1.616 (0.750-3.480)	3.082 (2.115-4.493)*	4.136 (2.448-6.989)*
**Number of traumatic events after the war**			
<4 events	1	1	1
≥ 4 events	0.679 (0.193-2.391)	5.769 (3.203-10.392) *	2.283 (1.065-4.964) *

*p <0.05.

We ran a separate multinomial analysis to determine similarities and differences in the combination of risk factors for depression only and PTSD only compared to risk factors for the comorbid diagnosis. Depression only was distinguished from PTSD-depression comorbidity by being severely disadvantaged, exposure to traumatic events during the war, and rural residency. PTSD only was differentiated from the comorbid condition by being severely disadvantaged and exposure to traumatic events after the Peace Agreement. By comparing the patterns of predictor variables for PTSD only and depression only, traumatic events during the war and after the Peace Agreement and rural/urban residency emerged as the variables distinguishing these two diagnoses (not displayed).

Table 4 shows the degree of psychological distress associated with each of the diagnostic categories. Results of one-way ANOVA showed that the differences in the mean GHQ score for each of the groups were statistically significant: individuals in the PTSD-depression comorbid group were likely to have a greater GHQ score (higher degree of psychological distress) compared to persons in the depression only or PTSD only groups.

**Table 4:** Result of one-way ANOVA: differences in psychological distress (measured by mean GHQ scoring) for four diagnostic groups

	**N**	**GHQ score, Mean (95% CI)**
Depression only	94	6.27 (5.47-7.08)
PTSD only	224	8.90 (8.61-9.37)
PTSD-depression comorbidity	89	11.00 (10.301-11.62)

F= 28.27, p <0.05.

Participants with PTSD-depression comorbidity did not differ significantly from those with PTSD only in their rate of exposure to traumatic events during the war (mean=7.8, CI (7.2-8.3) and mean=7.4, CI (7.1-7.8), respectively). The same tendency was found for exposure to traumatic events after the Peace Agreement (comorbid condition: mean=1.1, CI (0.6-1.6) and PTSD only: mean=1.6 CI (1.3-2.0)).

Compared to participants with PTSD only, participants with PTSD-depression comorbidity differed in some of the types of traumatic events they endorsed. Participants with a comorbid diagnosis, compared to those with PTSD only, had more frequently experienced lack of shelter (88.4% versus 79.3% [*X*^*2*^ =4.62, *p* <0.05]) and been physically harmed (59.8% versus 48.5% [*X*^*2*^ =4.30, *p* <0.05], during the war. The tendency was reversed for exposure to recent traumatic events (after the Peace Agreement). Participants with PTSD only reported a higher rate of the following events compared to those with PTSD-depression comorbidity: witnessing murder (14.6% versus 5.4% [*X*^*2*^ =6.67, *p* <0.05]) and murder or violent death of family member (12.2% versus 4.5% [*X*^*2*^ =5.47, *p* <0.05]).

## Discussion

In this study we aimed to examine the impact of socioeconomic disadvantages and exposure to traumatic events on PTSD-depression comorbidity, PTSD, and depression.

The significant association between socioeconomic disadvantage on both PTSD only and PTSD-depression comorbidity is noteworthy. Severe socioeconomic disadvantage was the risk factor with the strongest association to comorbid condition (OR= 8.096). Similarly, the results of our study revealed a dose–response association between exposure to traumatic events, on the one hand, and PTSD only and comorbid condition, on the other hand. Experiencing eight or more traumatic events increased the likelihood of having PTSD only or the comorbid condition. So did the exposure to four or more trauma after the Peace Agreement.

These findings are in agreement with the assumption that, rather than focusing merely on trauma exposure, the effect of socioeconomic conditions on mental disorders need to be emphasized in the post-conflict populations. Following the same line of argument, Miller and Rasmussen [15] distinguish between war exposure and ‘daily stressors’ as determinants of mental health in the population in war-effected settings. The authors emphasize the role of ‘daily stressors’ (stressful social and material conditions, ongoing adversity, or ecological stressors [50]), which directly and indirectly, influence the mental health. Acknowledging the role of a wider range of ecological-social factors can provide a better understanding of mental health outcome in the war-effected settings [18]. Although the term ‘daily stressor’ has been criticized for being imprecise which includes a variety of conditions and events [51], socioeconomic disadvantages in our study may be interpreted as ‘daily stressors’ for the participants.

Studies on the effect of ‘daily stressors’ in post-conflict populations imply that ‘daily stressors’ account for large proportion of mental distress [52-54]. Indeed, Miller and Rasmussen [17] argue that “level of exposure to daily stressors has consistently been a stronger predictor than direct war exposure on most mental health outcomes”. In Roberts et al.’s [26] recent study from the urban setting of Juba, South Sudan, variables related to living conditions (such as lack of food, water, and medical care) were associated with lower general physical and mental health. In our study severe socioeconomic disadvantage, in comparison to recent and older trauma exposure, showed a stronger association to PTSD-depression comorbidity. However, recent traumatic events, compared to severe socioeconomic disadvantage, showed greater association to having PTSD only. Although the rate of recent trauma was similar for PTSD only and comorbid groups, the PTSD only group, compared to the comorbid group, reported to have experienced a higher rate of more extreme and dramatic events such as witnessing murder or murder/violent death of family members.

The strong association between trauma exposure and severe socioeconomic disadvantage and comorbid condition, found in our study, adds to the existing knowledge in the field. What is noteworthy in addition, is the vulnerable position of participants with comorbid condition: not only they had experienced high level of traumatic events (comparable to those with PTSD only), but also their distress level (measured by GHQ-28) was significantly higher than those with PTSD only or depression only. A higher level of psychological distress associated with PTSD-depression comorbidity has also been reported in other studies of post-traumatic conditions [1]. For instance, amongst the survivors of an earthquake, psychological distress was reported to be significantly higher in the comorbid PTSD and depression group than in the PTSD only group (assessed using the GHQ-12) [55]. Another characteristic which distinguishes the comorbid group is the strong impact of trauma during the war (older trauma). Participants with history of trauma during the war were four times more likely to have a comorbid condition. Hence, health personnel should be particularly aware of the needs of persons with a comorbid diagnosis, and also of the characteristic pattern of risk factors.

The pattern of risk factors for the comorbid condition and PTSD only were similar to each other but different from pattern of risk factors for depression only. This finding provides some evidence to the notion that PTSD and comorbid PTSD-depression may be undistinguishable constructs. For instance, in O’Donnell et al.’s [9] study, PTSD and comorbid PTSD-depression emerged as undistinguishable constructs. However, this is not in accordance with other studies where the two conditions emerge as separate construct [56].

Living in a rural setting also emerged as a risk factor with different characteristics than living in an urban setting: residents of urban areas showed higher risk of having PTSD only and comorbid diagnosis, while rural residency was associated with having depression only. Generally, inequalities exist between rural and urban health care in Africa/low income countries both in regard to access to and utilization of services [57]. However, more research is needed on the South Sudanese context concerning urban/rural health and availability and utilization of mental health services. Being a returnee increased the odds of being in the PTSD only or the comorbid group. Returnees have been considered as a risk group in other studies [58].

The rate of PTSD and depression varies highly in different post-conflict populations [11]. For instance PTSD and depression was reported to be at 3.7% and 32.6% among population of postwar Jaffna in Sri Lanka [59], and 42% and 68% in Afghanistan [60]. The rate of PTSD in our sample was consistent with that of Roberts et al. [30] in Juba, South Sudan (37.6% and 36%, respectively). However, the rate of depression in our study was much lower than in Roberts et al.’s (16% and 50%, respectively). This discrepancy may be due to use of methods: Roberts et al. applied a screening symptom scale (the Hopkins Symptoms Check List-25) but in the present study a structured diagnostic interview was used in order to diagnose depression.

This study demonstrates that it was possible to conduct such a community survey under very difficult circumstances. For example, there was a lack of proper infrastructure, making it difficult to reach some of the sampling areas, and the security situation had to be carefully and continuously monitored. Emergency psychiatric treatment was therefore occasionally provided by the article’s fourth author (a physician). We believe the results, based on the randomized sample of the study, can be considered as generalizable for Greater Bahr el Ghazal States and relevant for other post-conflict settings. The findings, when related to other studies elsewhere in South Sudan [26,28-30], indicate extremely high levels of poor mental health in South Sudan.

This study also had some limitations. Being a cross-sectional study, it cannot provide a cause and effect relationship between the studied demographic and trauma exposure variables on the one hand and PTSD/depression on the other. Furthermore, we used self-report measures in order to assess exposure to traumatic events, which can pose bias in the form of inconsistency in the memory of events [61]. Self-reported measures rely on the participant’s memory and are also prone to be impacted by dominated attitudes toward the themes of study. The use of an additive scale of traumatic events is a simple way of including an indicator of exposure. However, it does not differentiate between the types and severity of the events. Furthermore, although the instruments used in this study have been widely used internationally in different cultural settings and the interviewers were familiar with the socio-cultural setting, no formal socio-cultural validation was conducted.

Living in a polygamous marriage was considered as a socioeconomic disadvantage because the participants in polygamous marriage were more likely to have no regular income, have low monthly income, be unemployed and have low level of education. It also showed unadjusted association with both depression only and comorbid condition (Table 2) (with no significant differences between male and female participants). However, cautions should be exercised about the social status of individuals in polygamous marriage as these may be perceived by the society as economically well off families who can offered a polygamous marriage.

We were not able to formally assess inter-rater reliability. However, attempt was made, through repeated and supervised interview practice, to ensure a satisfactory level of rating-agreement among the interviewers. Despite these limitations, our findings, based on data from a post-conflict setting, contribute to the ongoing debate on the relationship between PTSD and depression.

## Conclusions

We identified the PTSD-depression comorbid condition as having a risk profile which is similar to PTSD only, but distinct from depression.

Individuals with the comorbid condition are more vulnerable as they have been exposed to more traumatic events, are more likely to have sever socioeconomic disadvantages and show a higher level of associated psychological distress. A clinical implication of this finding is that persons exposed to traumatic events should be screened not only for PTSD but also for depression symptoms. The significant association of socioeconomic disadvantages advises against a one-sided focus on exposure to traumatic events in mental health studies performed in post-conflict settings. Considering the strong association of socioeconomic disadvantages and mental health of the population, the importance and urgency of general economic development in South Sudan becomes more salient.

The high rates of PTSD, depression, comorbid condition, and psychological distress, which also has been identified in other studies in South Sudan [26,28-30], combined with the immense lack of mental health services in the country, necessitates urgent attention. Although mental health services are included in the South Sudan’s Basic Package of Health services which is the main mechanism for delivering health care in the country [62], evidences of implementation of this plan is hard to detect. In addition to development of mental health curative facilities, systematic mental health promotion programs should be implemented.

Need assessment studies and further research on public attitude toward mental illness and the already existing strategies to deal with mental illness in the society are recommended.

Data from this study will be analyzed further and made available to the health authorities in South Sudan to be used in the further development of the health services in the country.

## Competing interests

The authors declare that they have no competing interests.

## Authors’ contributions

TA: executed the statistical analysis and drafted the manuscript; participated in the design of study. LL: participated in the design of study and drafting of the manuscript. AHE: participated in the design of study and drafting of the manuscript. MMR: participated in the design of the study and data collection. EH: supervised, participated in the design of study and drafting of the manuscript. All authors read and approved the final manuscript.

## Pre-publication history

The pre-publication history for this paper can be accessed here:

http://www.biomedcentral.com/1471-244X/12/175/prepub
